# EERS: Energy-Efficient Reference Node Selection Algorithm for Synchronization in Industrial Wireless Sensor Networks

**DOI:** 10.3390/s20154095

**Published:** 2020-07-23

**Authors:** Mahmoud Elsharief, Mohamed A. Abd El-Gawad, Haneul Ko, Sangheon Pack

**Affiliations:** 1 School of Electrical Engineering, Korea University, Seoul 02841, Korea; mahmoud@korea.ac.kr (M.E.); mgawad@korea.ac.kr (M.A.A.E.-G.); 2 Department of Computer Convergence Software, Korea University, Sejong 30019, Korea; heko@korea.ac.kr

**Keywords:** energy-efficiency, industrial wireless sensor networks, reference node selection, time synchronization

## Abstract

Time synchronization is an essential issue in industrial wireless sensor networks (IWSNs). It assists perfect coordinated communications among the sensor nodes to preserve battery power. Generally, time synchronization in IWSNs has two major aspects of energy consumption and accuracy. In the literature, the energy consumption has not received much attention in contrast to the accuracy. In this paper, focusing on the energy consumption aspect, we introduce an energy-efficient reference node selection (EERS) algorithm for time synchronization in IWSNs. It selects and schedules a minimal sequence of connected reference nodes that are responsible for spreading timing messages. EERS achieves energy consumption synchronization by reducing the number of transmitted messages among the sensor nodes. To evaluate the performance of EERS, we conducted extensive experiments with Arduino Nano RF sensors and revealed that EERS achieves considerably fewer messages than previous techniques, robust time synchronization (R-Sync), fast scheduling and accurate drift compensation for time synchronization (FADS), and low power scheduling for time synchronization protocols (LPSS). In addition, simulation results for a large sensor network of 450 nodes demonstrate that EERS reduces the whole number of transmitted messages by 52%, 30%, and 13% compared to R-Sync, FADS, and LPSS, respectively.

## 1. Introduction

Industrial wireless sensor networks (IWSNs) have been widely developed in the last decade. They play a significant role in many applications, e.g., industry, healthcare, agriculture, and smart metering systems. Currently, IWSNs perform as an essential foundation in the widespread industrial internet of things [1,2]. IWSNs typically consist of many sensor nodes that are spatially disseminated over a region of interest. Generally, sensor nodes are usually used to observe environmental conditions [3]. They are often battery-operated, and sometimes it is infeasible to recharge or replace batteries. Consequently, the lifetime of the battery becomes a crucial concern in the design of IWSNs [4,5]. To maximize the battery lifetime and conserve the battery power, a time-synchronous operation is regularly preferred. Most of the sensor nodes need time synchronization to coordinate wake up and sleep operations at an arranged time. Consequently, sensor nodes require trust and robust synchronization [5].

Time synchronization is considered as a critical part of the current industrial operation of sensor nodes. It offers a common reference time for whole sensor nodes in the network. Generally, sensor nodes are frequently prepared with hardware clock oscillator, which is relatively inexpensive, and inaccurate. In this way, it is much challenging to introduce precise synchronization for such sensor nodes [6,7]. With such sensor nodes, it is required to periodically conduct an accurate synchronization process to well-preserved time synchronization of networks for a long time [8]. Lack of synchronization in any sensor node can result in an imprecise wake-up time of the sensor node, which leads to a severe failure of network connectivity [9]. All sensor nodes, therefore, require regularly exchange timing messages in order to keep the whole network synchronized. Exchanging timing messages minimizes the time offsets that produced by the clock drift of each node in the network [7,10]. Frequent synchronization, however, dramatically raises energy consumption as the transmission of messages typically corresponds to the most significant portion of energy consumption [10].

In the literature, several conventional [11,12,13,14] and advanced [15,16,17] protocols for the synchronization of sensor nodes have been developed. The main aim of these protocols is to provide synchronization to all sensor nodes in networks. Most of these protocols were intended to focus on the accuracy of synchronization as it is very important with less concern of communication overhead. Besides, these protocols, due to frequent collisions, suffer from message loss. Over the past few years, a few synchronization protocols [5,6,18,19] have aimed to address the energy consumption issue. In general, however, the consideration gotten for the aspect of energy consumption is modestly lower contrasted to that for the synchronization accuracy [15]. We can conclude the energy consumption is a big challenge in the time synchronization of IWSNs. This challenge motivates us to introduce an energy-efficient reference node selection (EERS) algorithm for synchronization. It can substantially reduce the energy consumption by decreasing the number of transmitted messages among the nodes in the network. EERS minimizes the number of connected reference nodes and satisfies a minimum number of hops. Besides, EERS proposes a new method that avoids collisions completely among nodes during the time synchronization process. The contributions of EERS can be summarized as: (1) EERS significantly reduces the communication overhead (number of transmitting messages) and accelerates the reference scheduling, which can reduce energy consumption; (2) EERS employs a reference node scheduling method to resolve the collision problem and in turn to reduce power consumption; and (3) we implemented EERS in a real wireless sensor network with Arduino Nano RF sensors and conducted extensive large-scale simulations.

The remainder of this paper is organized as follows. We summarize the related works in Section 2. In Section 3, we elaborate on the system model and the operation of the EERS algorithm. In Section 4, we present the experimental and simulation results. The concluding remarks are finally given in Section 5.

## 2. Related Work 

Time synchronization for sensor nodes has been considerably analyzed for decades. Within the literature, there have been many methods for clock synchronization that are aimed toward minimizing energy consumption via decreasing the communication overhead and increasing the accuracy. In this section, we focus on those works which are concerned with reducing the communication overhead by decreasing the number of transmitted messages.

Noh et al. proposed the pairwise broadcast synchronization (PBS) [20]. It utilizes the pairwise operation introduced in [21]. PBS reduces the energy consumption (number of transmitted messages) by employing an overhearing technique in a single hop domain. The authors extended their work by introducing the Multi-hop PBS called groupwise pair selection algorithm [22]. It consists of a pair selection methods and hierarchy forming. GPA, however, needs an additional pairwise operation that reduces the energy efficiency compared with its single-hop counterpart [7]. Selecting pairs of nodes in a large network can be very costly in terms of both computation time and energy.

Spanning tree-based energy-efficient time synchronization (STETS) [23] has been proposed for the industrial Internet of things (IIoT). It employs the sender-to-receiver protocol (SRP) as well as receiver-to-receiver protocol (RRP). Especially in a large-scale and densely connected network, it effectively decreases energy consumption by reducing the number of transmitted messages. STETS, however, cannot synchronize all the nodes in some cases. The robust time synchronization (R-sync) [6] for IIoT has been introduced to resolve the drawbacks of STETS. Similar to STETS, R-Sync utilizes SRP and RRP. It focuses on identifying and pulling back the isolated nodes to the network that have lost their synchronization. Although R-sync requires relatively fewer numbers of messages compared to the timing-sync protocol for sensor networks (TPSN) [14] and STETS, it does not have any technique to avoid collisions. 

The energy-efficient coefficient exchange synchronization protocol (CESP) [19] was introduced by Gong et al. to address the excessive power consumption of the reference broadcast synchronization (RBS) [13]. CESP employs the synchronization coefficient to decrease the number of transmitted messages compared with RBS [10]. CESP, however, consumes relatively high power and does not have any technique to avoid collisions. Yildirim et al. proposed an adaptive value tracking synchronization (AVTS) [24] to resolve the drawback of quick flooding. AVTS provides a scalable synchronization and reduces memory overhead compared to the flooding time synchronization protocol (FTSP) [12] and flooding with clock speed agreement (FCSA) [25]. AVTS, however, uses the scheme of flooding messages which results in the low energy efficiency of the network.

The density table-based synchronization (DTSync) protocol [5] has been introduced. It utilizes the concept reference scheduling technique. Iteratively, the reference scheduling mechanism selects an ordered set of common nodes. These nodes are responsible for disseminating the timing messages in the entire network. Compared to hierarchy reference broadcast synchronization (HRTS) [11], DTSync requires fewer messages, which in turn minimize the energy consumption. Elsharief et al. introduced the fast scheduling and accurate drift compensation for time synchronization (FADS) [18]. Similar to DTSync, FADS employs a reference scheduling technique that organizes the message transmission among the sensor nodes. It improves the performance of the reference scheduling process by reducing the time consumption. FADS, however, during the reference scheduling process, produces a relatively large number of message broadcasts which causes excessive energy consumption [10].

Recently, we proposed a low power scheduling for time synchronization protocols (LPSS) [10]. Compared to FADS, LPSS can significantly reduce the number of broadcasted messages and accelerates the reference scheduling process in a centralized manner, which in turn reduces the energy consumption. Besides, it provides a scheme to avoid collisions. LPSS, however, selects reference nodes randomly at each level which leads to the inefficient energy usage of the network. 

## 3. EERS Algorithm

In this section, we first describe the system model for the EERS algorithm and detail the operation of the EERS algorithm.

### 3.1. System Model

In this paper, a wireless sensor network is designed as an undirect graph G=(V, E) consisting of a set of sensor nodes, V={1, 2,….,…, N}, in the network and a set of links, E ⊆V×V, indicating the connection among the sensor nodes. That is, (i,j)∈E if node i  and node j are located within the transmission range of each other [26]. In this paper, each node has a unique ID number and is supplied with a hardware clock oscillator. For simplicity, we assume that the network is stationary, and the transmission among nodes is reliable. Besides, it is assumed that all sensor nodes are identical (i.e., they have the same transmission range of R meter radius). Moreover, we assume that the sink node recognizes the position of every node in the network.

### 3.2. Operation of EERS 

EERS is a greedy heuristic algorithm that attempts to minimize the communication overhead. It guarantees the coverage of all nodes and accelerates the reference node scheduling in a tree-based network topology. Additionally, to guarantee collision avoidance during the synchronization process, EERS assigns an exclusive scheduled time slot to each reference node. 

The pseudo-code of EERS is presented in Algorithms 1 and 2. Algorithm 1 is used by the sink node only. It describes the process of selecting reference nodes, RefNodes, and their scheduled time slots, SchSlots. On the other hand, Algorithm 2 is used by other nodes in the network, which shows the procedure of receiving the RefNodes and SchSlots. 

In the beginning, in Algorithm 1, to guarantee the minimum hops starting from sink node to farthest node in the network, sink node has perfect awareness of the network topology and utilizes the breadth-first search (BFS) [27] algorithm to determine the level of every node in the network (Algorithm 1, Line 5). As a result of BFS, we have a tree-based network consisting of L levels. Each level, k, has a set of nodes, $N_{k}$. The optimal aim is to find the minimum number of connected reference nodes. In fact, finding a minimum number of reference nodes is a non-deterministic polynomial-time (NP)-hard problem [4,26]. Specifically, its time complexity is $O\left(m\times m!\right)\leq O\left(m^{m}\right)$, where m is the number of nodes in each level. In this article, our target is finding a simple approximation algorithm to find the minimum set of reference nodes, $R_{k}\subseteq \;N_{k}$, that cover all nodes in the next level, k+1. At the end of the initial step, the following steps are sequentially conducted. 

***Step 1:*** As the sink node, S, knows the position of each node, it determines the distance between each node in the level k and its neighbor nodes in the level k+1 (Algorithm 1, Lines 7–9). The distance, $d_{i_{k,\;}j_{k+1}}$, between node $i_{k}\in N_{k}$, and node $j_{k+1}\in N_{k+1}$ is calculated as follows

(1) $$d_{i_{k,\;}j_{k+1}}=\sqrt{{\left(x_{i_{k}}-x_{j_{k+1}}\right)}^{2}+{\left(y_{i_{k}}-y_{j_{k+1}}\right)}^{2}}$$

where $\left(x_{i_{k}},y_{i_{k}}\right)$ and $\left(x_{j_{k+1}}\;,\;y_{j_{k+1}}\right)$ are the position of the nodes $i_{k}$, and $j_{k+1\;}$, respectively. As a result of this process, EERS builds a set of the distances between $N_{k}$ and $N_{k+1}$, $d_{N_{k+1}}^{N_{k}}$.
**Algorithm 1:** EERS pseudo-code for reference node selection (sink node only)Initial:,CoverNodesFlag←false,cnt ←0, k←0, SeqN←01.s←sink node2.RefNodes(1)←s3.Schslot(1)←04.CoverNodesFlag(s)←true5.Compute the hop distance from s and the level to every node (i.e., using BFS)6.while (k !=LevelMax−1) do**//**LevelMax is equal to the level of the farthest node from sink node 7.  
$for\;\left(every\;edge\;\left(i,j\right)\in E,\;i\in N_{k},j\in N_{k+1}\;\;\right)\;do$
8.    
$\mathrm{detrmine}\;d_{i,j}$
9.  
end for
10.  
$while\;\left(CoverNodesFlag\left(N_{k+1}\right)\;!=true\right)\;do$
11.     
$R\leftarrow N_{k}\left(\max\left(d_{i,j}\right)\right)$
12.     
RefNodes(cnt)←R
13.     
SchSlots(cnt)←cnt
14.     CoverNodesFlag(m) ←true  // $\left(m\subseteq \;N_{k+1}\right)$
are the neighbor nodes of R
15.     
cnt←cnt+1
16.  
end while
17.k←k+118.end while19.SeqN←SeqN+120.brodcast refernces scheduling message <RefNodes,SchSlots, SeqN> 

***Step 2:***S  selects the reference node, R using the equation (Algorithm 1, Line 11)

(2) $$R=N_{k}\left(Max\left(d_{N_{k+1}}^{N_{k}}\right)\right)$$

where the function $Max\left(d_{N_{k+1}}^{N_{k}}\right)$ chooses a node that can cover the farthest node in the k+1 level. 

Then, EERS adds R  to RefNodes. Next, S assigns a scheduled time slot to R and adds it to SchSlots (Algorithm 1, Lines 12 and 13). 

***Step 3:*** To avoid processing a node in $N_{k+1}$ more than once, we mark it as a covered node (Algorithm 1, Line 14). Then, we update the $d_{N_{k+1}}^{N_{k}}$ by removing the distance between it and other nodes in $N_{k}$. 

***Step 4:***S increments the sequence number, SeqN (Algorithm 1, Line 19). SeqN is added to prevent sending the scheduling message many times. Next, S broadcasts the references scheduling message among all nodes in the networks (Algorithm 1, Line 20).

***Steps 1***–***4*** in Algorithm 1 are repeated until no more reference node can be chosen in the network. We can conclude the time complexity of EERS is $\sum_{k=0}^{LevelMax-1}O\left(\;N_{k}\right)\;\approx O\left(N\right)$ where LevelMax is the highest level in the network. 

Upon receiving the scheduling message (Algorithm 2), each node only retracts if the received sequence number is larger than the stored one (Algorithm 2, Line 2). Next, the node checks if its ID has been recorded in RefNodesl then, it keeps the corresponding time slot, mySchslot (Algorithm 2, Lines 5–7). Next, every reference node initiates the waiting timer (WT). The value of WT can be computed as
**Algorithm 2:** EERS pseudo-code for reference scheduling selection (except sink node).Initial:cnt ←0,SeqN ←01.◼ **Upon receiving**
Scheduling Message
2.if (Received SeqN>SeqN) Then3. 
SeqN ←Received SeqN
4. 
for (cnt ←0 to length(RefNodes))do
5.  
if (NodeID==RefNodes(cnt)) Then
6.   
mySchsSlot←  SchSlots(cnt);
7.  
end if
8.  
if (RefNodes(cnt)==SenderID) Then
9.   
$SchSlot_{SrcID}\leftarrow \;\;SchSlots\left(cnt\right)$
10.  
end if
11. 
end for
12. 
$WaitingTime\;\leftarrow \left(\;mySchSlot-SchSlot_{SrcID}\right)$
13. Setup waiting timer WT(WaitingTime)14.end if15.◼ **Upon**
WT
**expires**16.forward refernces scheduling message <RefNodes,SchSlots, SeqN. >

(3) $$WTvalue=mySchslot-Schslot_{SrcID}$$

where WTvalue is the time that each reference should wait before forwarding the scheduling message, $Schslot_{SrcID}$ is the sender’s time slot, and mySchslot is the current reference time slot (Algorithm 2, Line 12). As soon as WT expires, the current reference forwards the scheduling message to its neighbor nodes (Algorithm 2, Lines 15 and 16). 

**Figure 1:**
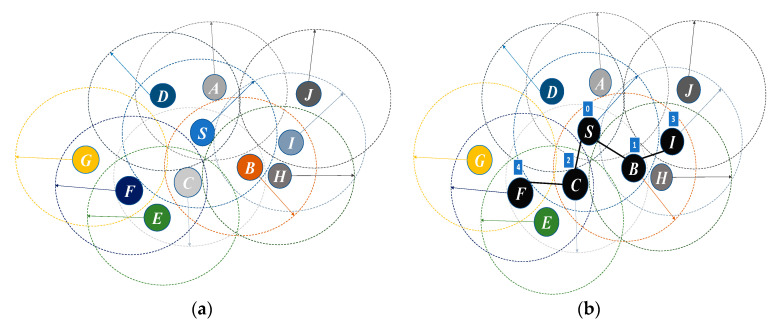
Wireless sensor network example. (**a**) A sensor network before applying EERS. (**b**) The EERS chooses the reference nodes (colored in black) and assigns schedule time slot to each reference.

Figure 1 shows an example of EERS. Node S  applies Algorithm 1 to select reference nodes (see Figure 1a) and assigns an exclusive scheduled time slot to each reference (see Figure 1b). Upon receiving the reference scheduling message, each node applies Algorithm 2 to verify if it has been selected as a reference node. For example, in Figure 1, it is assumed that node F is selected as a reference node and its assigned scheduled time slot is 4. As soon as it receives a packet form node C, it determines its waiting time equals to 2 (=4 (i.e., F’s mySchSlot) – 2 (i.e., $Schslot_{SrcID}$ of node C)). Then, F sets up its waiting timer and it forwards the received message to its neighbor nodes upon the WT is expired. 

## 4. Experimental and Simulation Results

In this section, we present experimental results achieved using a network of real hardware sensors. EERS was implemented in a C++ program and ported on an embedded processor in the hardware sensors. Next, we performed experiments with wireless networks composed of each hardware sensor running the C++ program. In addition, we compared our algorithm with the previous methods, FADS [18], LPSS [10], and R-Sync [6]. Note that the synchronization protocol in [5] was used for EERS to highlight the benefit of EERS. 

To show the performance of EERS for a large-scale network, we also conducted a simulation study and compared the performance of EERS with FADS, LPSS, and R-Sync. In our experimental and simulation studies, the number of transmitted messages was quantitatively analyzed, and the time of the whole procedure was measured, as they have a significant impact on energy consumption. Generally, the wireless network is a broadcast domain. For each transmission, therefore, there are multiple receptions [28]. For example, assume that we have a uniformly distributed network with N nodes, and each node has B surrounding nodes. The total energy consumption of the transceiver, $E_{T},$ can be expressed by transmission energy $\left(E_{TX}=A.P_{TX}.T_{TX}\right)$, and reception energy $\left(E_{RX}=A.B.P_{RX}.T_{RX}\right)$ as defined by the following equation.

(4) $$E_{T}=A(P_{TX}.T_{TX}+B.P_{RX}.T_{RX})$$

where A is the number of transmitted messages, B is the number of received messages, $P_{TX}$ is the transmission power, $P_{RX}$ is the reception power, $T_{TX}$ is the time duration of the transmitted packet, and $T_{RX}$ is the time duration of the received packet. For the sake of simplicity, consider $P_{TX}\approx P_{TX}=P,$ the packet length, L, and data rate, D, are fixed. Hence, $T_{TX}=T_{RX}$=$\;\frac{L}{D}$. We can, therefore, simplify Equation (4) as Equation (5).

(5) $$E_{T}=A\frac{P.L\left(B+1\right)}{D}$$

From Equation (5), it can be noted that the energy consumption is directly proportional to the number of transmitted messages. Besides, reducing the number of messages decreases the time it takes for the transmitter node to transmit all its messages. This in turn allows the node to spend less time in idle mode while processing the transmission procedure, and to switch to sleep earlier. Since the idle mode also consumes energy [19], minimizing idle time can further save energy. Considering the transceiver of Arduino Nano RF [29], we conducted a wireless network of 25 nodes. Each node in the network can communicate with each other. Therefore, the total energy consumption per each transmission is equal to $\frac{49\times 8\times \left(33.9m+24\times 36.9m\right)}{250k}\approx 1.44\;mJ$. 

### 4.1. Experiments Setting 

**Figure 2:**
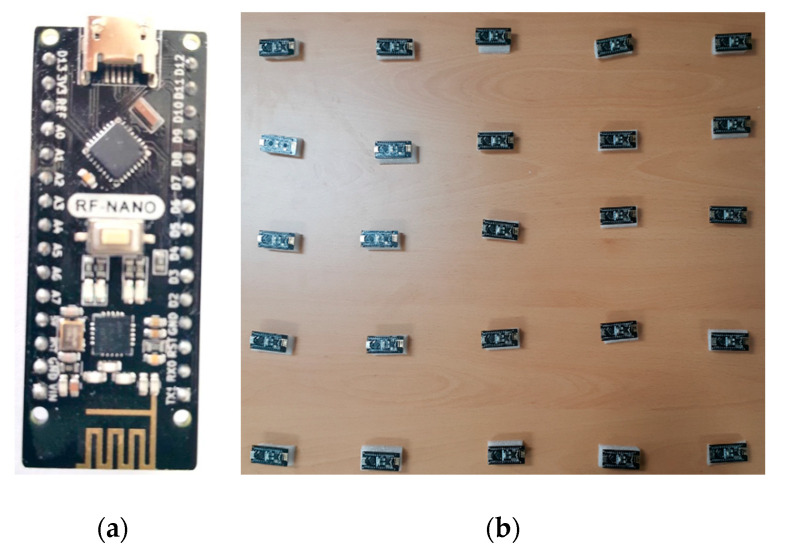
(**a**) Hardware platform (**b**) Set up of a network of 25 sensor nodes for the experiment.

**Figure 3:**
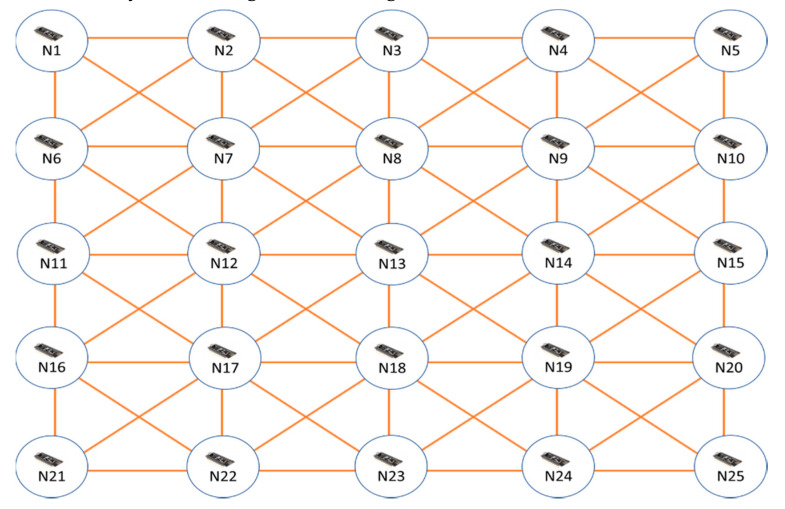
Sensor node in 4-way grid topology.

In general, choosing a sensor module is dependent on the application’s requirements (computing power, power consumption, memory, dedicated range, data rate, etc.) and the budget. Here, in this paper, our target is spreading the timing messages over a multi-hop network for smart metering applications. We chose an Arduino Nano RF [29] to implement the hardware for sensor nodes. The hardware integrates an ATmega328P CPU core [30] and an NRF24L01 Rf transceiver on-chip [31]. Arduino Nano RF is a cheap module with acceptable memory, data rate, and transmission range. However, EERS can be implemented on other types of sensors modules. Table 1 provides the specification of the sensor board. A photo of the sensor board is shown in Figure 2a. We adopted a wireless sensor network of 25 nodes, as displayed in Figure 2b. We configured a network of the four-way grid topology illustrated in Figure 3 to test EERS over a multi-hop network. For simplicity of the testing, all the nodes were configured with a preconfigured set of neighbor nodes and the distances among them. The solid lines in Figure 3 indicate such neighbor nodes assigned to every node. The sensor nodes can communicate with each other if they have a common solid line. With this experiment, the performance of EERS could be evaluated under realistic conditions of congestion, transmission contention, and various packet collisions. Even though we did the tests using a simple network of small size in the laboratory, these tests demonstrated that EERS can scale to networks of a large scale with longer wireless range and can provide equally great execution. 

### 4.2. Experimental Results

This section describes the evaluation of the EERS, LPSS, FADS, and R-sync protocols. First, we evaluate the reference node scheduling process. R-sync is excluded from this evaluation as it does not have any reference scheduling mechanism. In the reference node scheduling procedure, the key performance indicator is the number of transmitted messages and the consumed time of the whole process. Table 2 shows that EERS has considerably fewer messages than LPSS and FADS for the scheduling process. For the network topology of Figure 3, EERS sent only 11 messages, while LPSS sent 17 messages (around 1.5 times more) and FADS exchanged 24 messages (around two times more). Next, for time consumed by the reference node scheduling procedure, the experimental results show EERS is faster than that of LPSS and FADS. Table 2 also compares the processing time measured for EERS, LPSS, and FADS protocols. EERS can reduce the whole time of the process to around 35% and 76% compared to LPSS and FADS, respectively. 

Finally, we applied the synchronization protocol of [5] on top of the EERS. The experimental results show that EERS and FADS transmit fewer messages than other protocols for the synchronization process. Table 2 shows that EERS has considerably fewer messages than LPSS and R-Sync. EERS and FADS required around 27 messages, while LPSS exchanged 45 messages (around 1.66 times more) and R-Sync exchanged 51 messages (around 1.88 times more). 

### 4.3. Large-Scale Simulation Results

Experimental results reveal that EERS improves energy consumption in a small test network. To further validate EERS, we developed a simulator that conducts the algorithms of EERS, LPSS, and FADS. Meanwhile, the simulation results of R-Sync were adopted from [6]. Simulations were developed in MATLAB using wireless networks of various sizes, and different transmission ranges. We presumed there are N sensor nodes which are randomly distributed in a square area of 1000 m × 1000 m. We also assumed that all nodes are identical and independent. Key simulation parameters are summarized in Table 3. In fact, we adopted most of the simulation parameters from [6]. We believe the simulation parameters is an appropriate choice to simulate realistic scenarios. By variation of the transmission range, we could simulate many types of sensor nodes which have different wireless ranges. For example, Arduino Nano RF has up to 100 m radius, which is located between 85 and 160 m (simulation range). On the other hand, changing the number of nodes in the network allowed us to demonstrate the behavior of our protocol in the sparse network (200 nodes) up to the dense network (450 nodes). We focused on the number of transmitted messages and the time of the process to show the performance of EERS. To reduce the impact of random errors, we ran 10,000 cycles for each experiment and obtained their average value. To illustrate the effect of the position of the sink node on the simulation results, two positions of sink node were tested: (1) sink node located at the center of the network; and (2) sink node located at the corner of the network. As expected, there is no big difference in the results due to the uniform distribution of the nodes. Therefore, to avoid redundancy, we only report the results in which the sink node was located at the center of the network.

**Figure 4:**
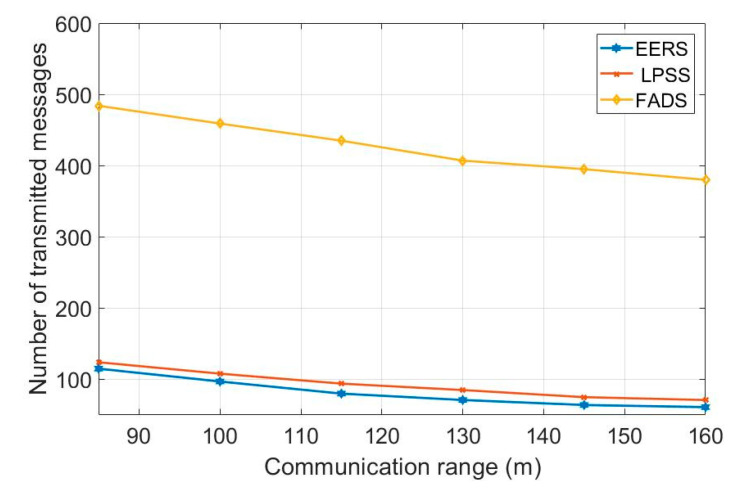
Required number of messages for reference node scheduling process (r = 85 m: 160 m, N = 240).

**Figure 5:**
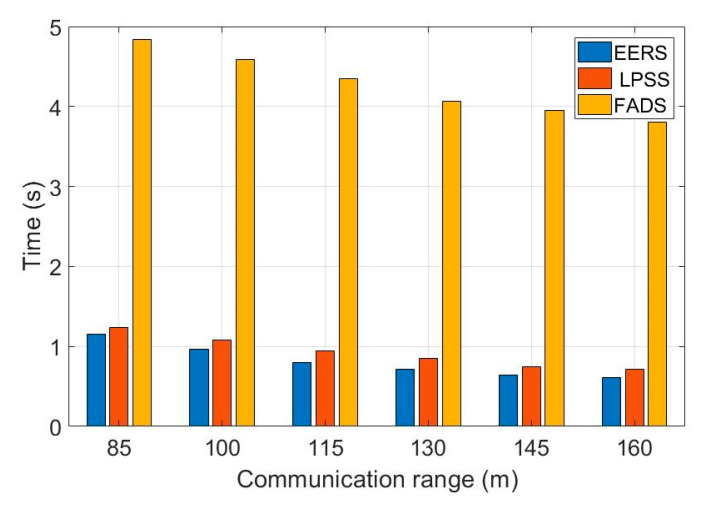
Required time for reference node scheduling process (r = 85 m: 160 m, N = 240).

**Figure 6:**
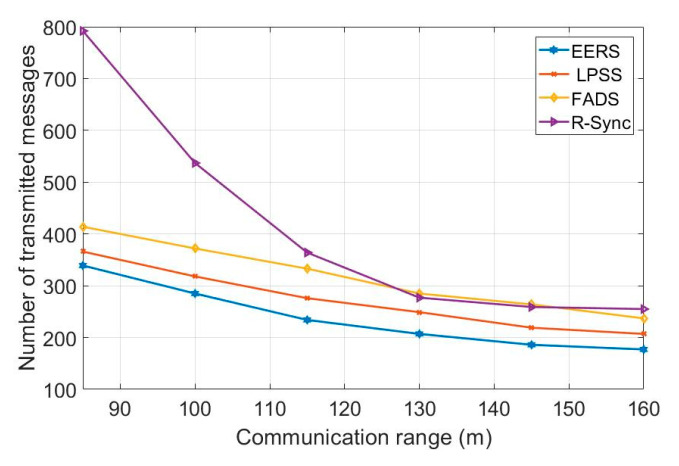
Required number of messages for synchronization process (r = 85 m: 160 m, N = 240).

**Figure 7:**
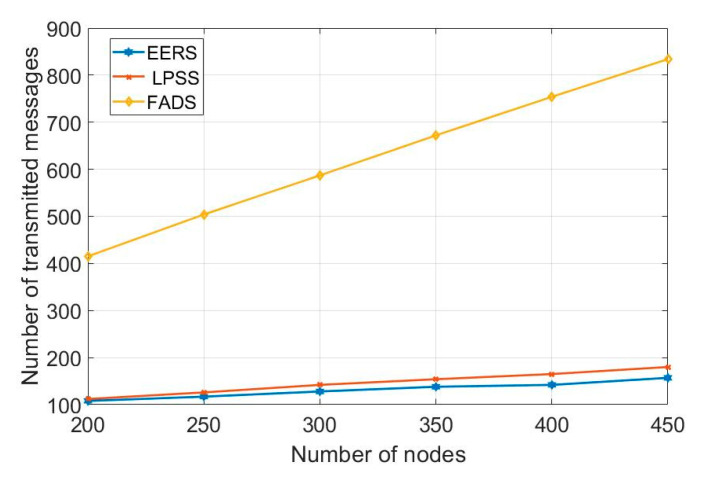
Required number of messages for reference node scheduling process (r = 85 m, N = 200: 450).

**Figure 8:**
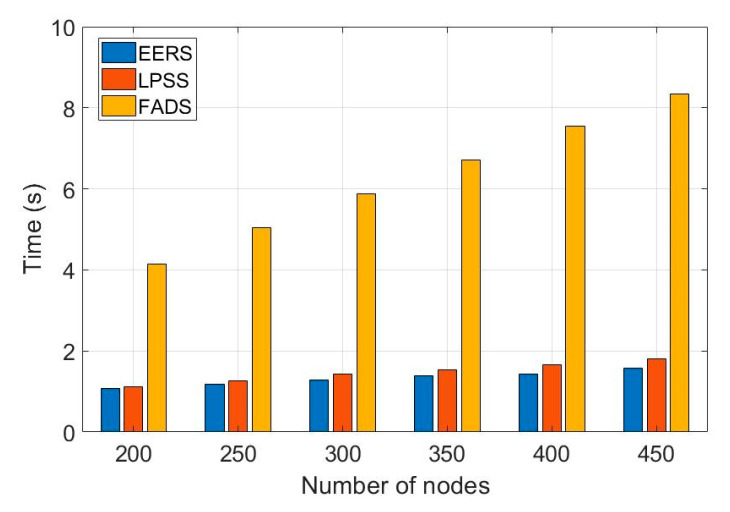
Required time for reference node scheduling process (r = 85 m, N = 200: 450).

**Figure 9:**
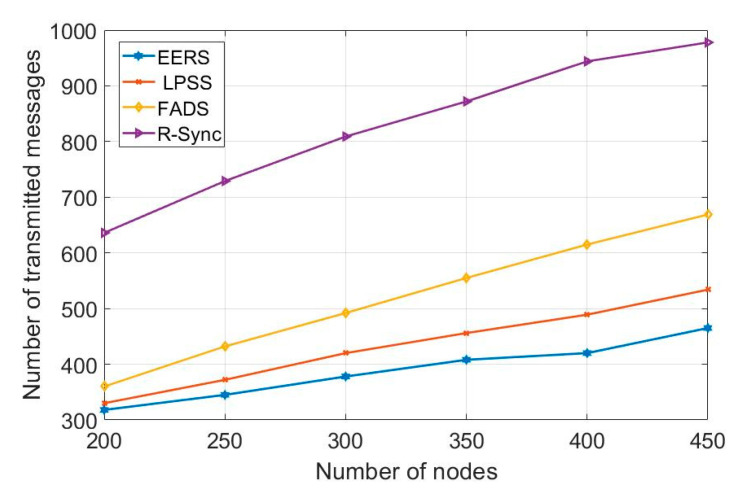
Required number of messages for synchronization process (r = 85 m, N = 200: 450).

First, we evaluate the performance of all protocols (i.e., EERS, LPSS, FADS, and R-Sync) under transmission ranges from 85 to 160 m. Figure 4 shows the number of transmitted messages at the reference node scheduling process while Figure 5 shows the time of the whole process. For a network with the transmission range of 160 m, EERS, LPSS, and FADS generate 61, 71, and 380 messages, respectively. EERS outperforms LPSS and FADS by factors of 1.2 and 6.3, respectively. On the other hand, EER can decrease the time of the scheduling process to around 14% and 84% compared to LPSS and FADS, respectively, as shown in Figure 5. In the synchronization process. EERS has considerably fewer messages than LPSS, FADS, and R-Sync, as depicted in Figure 6. EERS outperforms LPSS, FADS, and R-Sync by factors of 1.17, 1.34, and 1.44, respectively. Next, we evaluate the performance of EERS, LPSS, FADS, and R-Sync over various network sizes from 200 to 450 nodes. Figure 7 shows the number of transmitted messages at the reference node scheduling process. For a large network with 450 nodes, EERS, LPSS, and FADS generate 157, 180, and 834 messages, respectively. EERS outperforms LPSS and FADS by factors of 1.15 and 5.3, respectively. On the other hand, EER can decrease the time of the scheduling process to around 13% and 81% compared to LPSS and FADS, respectively, as shown in Figure 8. In the synchronization process, for a large network with 450 nodes, EERS has considerably fewer messages than LPSS, FADS, and R-Sync, as depicted in Figure 9. EERS outperforms LPSS, FADS, and R-Sync by factors of 1.15, 1.4, and 2.1, respectively. 

From the above results, it can be concluded that EERS consumes less energy than other techniques. At the same time, EERS does not lose the accuracy of the timing. Additionally, EERS is well-suited for those applications in which the position of each node is recognized, e.g., smart metering. Further, without losing its properties, EERS can handle high-density networks and therefore it can be considered as a highly scalable and adaptive protocol. 

## 5. Conclusions

In this paper, we consider the recent synchronization protocols for wireless sensor networks and introduce the energy-efficient reference node selection algorithm for synchronization in IWSNs (EERS). EERS resolved major drawbacks of prior schemes (i.e., LPSS, FADS, and R-Sync) by introducing an efficient reference node scheduling process. EERS significantly reduced the number of messages in both scheduling and synchronization process, which leads to large savings in energy consumption. On the other hand, it accelerates the scheduling process. We conducted extensive simulations as well as the real measurement with hardware sensor nodes of the EERS algorithm. The experimental results show that EERS requires fewer messages than previous methods such as LPSS, FADS, and R-Sync. 

As our future work, we are interested in combining deep learning techniques with EERS to find the optimal minimum set of reference nodes. The graph convolutional network will estimate the likelihood for each node in the graph to determine whether this node is part of the optimal solution [32]. Additionally, we plan to further improve EERS by allowing some of the reference nodes at the same level exchanging timing messages using the same scheduled time slot.

## Figures and Tables

**Table 1:** Specification of sensor module.

Parameter	Value
Carrier frequency	2.4 GHz
Memory	2 KB RAM, 32 KB Flash
Bit rate	250 Kbps
Transmission power	33.9 mw
Reception power	36.9 mw
Modulation type	GFSK
TX power	0 dBm
Frame size	49 Bytes
Scheduled time slot	10 ms
Crystal oscillator frequency	16 MHz

**Table 2:** Comparison of EERS, LPSS, FADS and R-sync.

	Protocol	EERS	LPSS	FADS	R-Sync
Operation					
Scheduling process (message)		11	17	25	-
Time of scheduling process (second)		1.1	1.7	46	-
Synchronization process (message)		27	45	27	51

**Table 3:** Simulation Parameters.

Parameter	Value
Network area	1000 m ×1000 m
Networks size (N)	200, 250, 300, 350, 400 and 450 nodes
Nodes distribution	uniformly distribution
Node wireless range radius (r)	85, 100, 115, 130, 145, and 160 m
Scheduling time width	10ms
Sink node position	Center (500 m, 500 m), Corner (0,0)
